# Supplementary material for: Mathematical model of a personalized neoantigen cancer vaccine and the human immune system
Source: PLoS Comput Biol. 2021 Sep 24;17(9):e1009318. doi: 10.1371/journal.pcbi.1009318 (PMC8462726; doi:10.1371/journal.pcbi.1009318)
Supplement: S1 Table — Mathematical model parameter definitions, value ranges, and values used in simulations. (PDF) [file pcbi.1009318.s002.pdf]

S1 Table. Parameter values and definition

S1 Table. Parameter values and definition.(♣ estimated from literature; ♦ estimated from data (clinical/experimental), see S1 Appendix for details; ★ fitted to trial data)

	Parameter	Definition	Value/Range	Value Used	Units
**Vaccine**	$Dose_p$	Peptide concentration at each dose	clinical trial data [1]	patient-specific ♦	pmol
	$Dose_a$	Adjuvant concentration at each dose		500 [1,2]	mg/L
	α_p	Internalization rate of peptides by DCs		0.28 [3]	day^{-1}
	α_d	Internalization rate of adjuvant by DCs		0.5	day^{-1}
	V_{sc}	Volume of injections site	-	$4 \times (0.1919)$ [4]	L
**Dendritic cells**	Λ	Maximum growth rate		3.75	day^{-1}
	δ_M	Death rate of mature DCs	(0.23,0.54) [5]	0.33	day^{-1}
	r_D	Maximum differentiation rate		2.48 ★	day^{-1}
	K_a	Half-maximum adjuvant effect constant		6.64 ★	-
	K_{DC}	Carrying capacity	$(2.07, 2.68) \times 10^7$ ♦	2.38×10^7 (median)	cells
	V_E	Volume of endosomes in a DC	$(0.16, 1.35) \times 10^{-14}$ [6]	1×10^{-14}	L
**Antigen processing and presentation**	N_A	Avogadro's constant	-	6.02×10^{23}	mol^{-1}
	α_p^E	Endosomal internalization rate of peptides	[10.8,144] [6]	70	day^{-1}
	β_p	Degradation rate of peptides	14.4-21.6 [7]	14.4	day^{-1}
	$k_{\text{on},1}$	On rate for T-epitope-MHCI binding with allele j		1.8144×10^{-2} [8]	$\text{pM}^{-1}\cdot\text{day}^{-1}$
	$k_{\text{off},j}$	Off rate for T-epitope-MHCI binding with allele j	Neoantigen specific (based on $K_{D,j}^{\text{eff}}$)	$k_{\text{on},1} \cdot K_{D,j}^{\text{eff}}$	day^{-1}
	$k_{\text{on},2}$	On rate for T-epitope-MHCII binding with allele k	$[0.43, 4.3] \times 10^{-2}$ [6,9]	8.64×10^{-3} ♦	$\text{pM}^{-1}\cdot\text{day}^{-1}$
	$k_{\text{off},k}$	Off rate for T-epitope-MHCII binding with allele k	Neoantigen specific (based on $K_{D,k}^{\text{eff}}$)	$k_{\text{on},2} \cdot K_{D,k}^{\text{eff}}$	day^{-1}
	β_M	Degradation rate of endosomal free MHCI/II molecules	0.92-2.08 [6]	1.663 [6]	day^{-1}
	k_{in}	Recycling rate of free MHC molecules	10.8-14.4 [6,7]	14.4 [6]	day^{-1}
	k_{ext}	Exocytosis rate of p-MHC complex	17.28-43.2 [6,7]	28.8 [6]	day^{-1}
	β_{pM}	Degradation rate of p-MHCI/II complex	≥ 0.1663 [6]	0.166 [6]	day^{-1}

Continued on next page

Table 1 – continued from previous page

	Parameter	Description	Value/Range	Value Used	Units
**T-cells**	a	Number of tumor cells needed for half-maximal A_{CD8} proliferation		5×10^7 [10]	cells
	a_1	Half saturation constant of the $CD4^+$ T-cells production rate		1×10^3 [10–12]	cells
	b_4	Max. growth rate of naïve $CD4^+$ T-cells		0.15 [13]	day^{-1}
	b_8	Max. growth rate of naïve $CD8^+$ T-cells		0.12 [13]	day^{-1}
	σ_4	Max. activation rate of naïve $CD4^+$ T-cells		1.5 [6, 14]	day^{-1}
	σ_8	Max. activation rate of naïve $CD8^+$ T-cells		3 [14]	day^{-1}
	K_{TC}	Carrying capacity of T-cells		8.57×10^{11} ♣	cells
	F_{P_4}	Frequency of antigen-specific $CD4^+$ T-cells	(0,0.01) [1]	$\{0.006, 0.001, 0.001, 0.001, 0.003, 0.001\}$	-
	F_{P_8}	Freq. of antigen-specific $CD8^+$ T-cells	(0,0.01) [1]	$\{0.007, 0.002, 0.006, 0.002, 0.002, 0.001\}$	-
	K_{pM}	Half-maximum effect of activation	400 [3, 6]	400	-
	c	Maximum $CD8^+$ T-cells recruitment rate		patient-specific★	day^{-1}
	c_4	Maximum $CD4^+$ T-cell production rate		patient-specific★	day^{-1}
	c_8	Rate at which $CD8^+$ T-cells are stimulated to be produced		6.5×10^{-11} [12, 15, 16]	day^{-1}
	μ	Death rate of naïve T-cells	(0.00014,0.012) [13, 17]	0.0029	day^{-1}
	μ_4	Death rate of activated $CD4^+$ T-cells	(0.022,0.083) [18]	0.031	day^{-1}
	μ_8	Death rate of activated $CD8^+$ T-cells	(0.022,0.052) [18]	0.022	day^{-1}
	ρ_4	Proliferation rate for activated T-cells	(0.0053,0.0477) [13]	0.0265	day^{-1}
	ρ_8	Proliferation rate for activated $CD8^+$ T-cells	(0,0.1106) [13]	0.0509	day^{-1}
**Tumor cells**	r	Maximum growth rate	tumor-type dependent	0.004 ♣	day^{-1}
	K_T	Carrying capacity of tumor		1.45×10^{10} ⧫	cells
	d	Max. lysis rate by activated T-cells	(0.01,0.05) [19]	patient-specific★	day^{-1}
	λ	Dependence of lysis rate on the effector/target ratio constant	(0,1)	patient-specific★	-
	s	Half-maximal effect of tumor cell lysis		0.0839 [12]	-

Continued on next page

Table 1 – continued from previous page

	Parameter	Description	Value/Range	Value Used	Units
**Initial Conditions**	$A_d(0)$	Adjuvant concentration in a vaccine		500	mg/L
	$p(0)$	Peptide amount in a vaccine		patient-specific ♦	pmol
	$D_I(0)$	Immature dendritic cells		1×10^7	cells
	$D_M(0)$	Mature dendritic cells		0	cells
	$p^E(0)$	Endosomal peptides		0	pmol
	$M_j^E(0)$	Endosomal MHC-I		$(1.6, 5.8) \times 10^{-7}$ [3,6]	pmol
	$M_k^E(0)$	Endosomal MHC-II		$(0.16, 8.3) \times 10^{-8}$ [3,6]	pmol
	$pM_j^E(0)$	Endosomal p-MHCI complex		0	pmol
	$pM_k^E(0)$	Endosomal p-MHCII complex		0	pmol
	$pM_j(0)$	p-MHCI on DC membrane		0	pmol
	$pM_k(0)$	p-MHCII on DC membrane		0	pmol
	$M_j(0)$	Free MHC-I on DC membrane		0	pmol
	$M_k(0)$	Free MHC-II on DC membrane		0	pmol
	$N_{CD4}(0)$	Naïve $CD4^+$ T-cell count	$(2.15, 8.6) \times 10^9$♦	$(5.38 \times 10^9) \times 0.7$	cells
	$N_{CD8}(0)$	Naïve $CD8^+$ T-cell count	$(2.15, 8.6) \times 10^9$♦	$(5.38 \times 10^9) \times 0.3$	cells
	$A_{CD4}(0)$	Activated $CD4^+$ T-cell count	patient-specific ♦		cells
	$A_{CD8}(0)$	Activated $CD8^+$ T-cell count	patient-specific ♦		cells
	$T(0)$	Tumor cell count		patient-specific ♦	cells

References

1. Ott PA, Hu Z, Keskin DB, Shukla SA, Sun J, Bozym DJ, et al. An immunogenic personal neoantigen vaccine for patients with melanoma. Nature. 2017;547(7662):217–221. doi:10.1038/nature22991.

2. Keskin DB, Anandappa AJ, Sun J, Tirosh I, Mathewson ND, Li S, et al. Neoantigen vaccine generates intratumoral T cell responses in phase Ib glioblastoma trial. Nature. 2019;565(7738):234–239. doi:10.1038/s41586-018-0792-9.

3. Chen X, Hickling T, Vicini P. A mechanistic, multiscale mathematical model of immunogenicity for therapeutic proteins: part 1—theoretical model. CPT: pharmacometrics & systems pharmacology. 2014;3(9):1–9.

4. Tegenge MA, Mitkus RJ. A physiologically-based pharmacokinetic (PBPK) model of squalene-containing adjuvant in human vaccines. Journal of pharmacokinetics and pharmacodynamics. 2013;40(5):545–556.

5. Lanzavecchia A, Sallusto F. Regulation of T cell immunity by dendritic cells. Cell. 2001;106(3):263–266.

6. Yogurtcu ON, Sauna ZE, McGill JR, Tegenge MA, Yang H. TCPro: an In Silico Risk Assessment Tool for Biotherapeutic Protein Immunogenicity. The AAPS journal. 2019;21(5):96.

7. Singer DF, Linderman JJ. The relationship between antigen concentration, antigen internalization, and antigenic complexes: modeling insights into antigen processing and presentation. The Journal of cell biology. 1990;111(1):55–68.

8. Corr M, Slanetz A, Boyd L, Jelonek M, Khilko S, al Ramadi B, et al. T cell receptor-MHC class I peptide interactions: affinity, kinetics, and specificity. Science. 1994;265(5174):946–949. doi:10.1126/science.8052850.

9. Kasson PM, Rabinowitz JD, Schmitt L, Davis MM, McConnell HM. Kinetics of peptide binding to the class II MHC protein I- Ek. Biochemistry. 2000;39(5):1048–1058.

10. Hu X, Jang SRJ. Dynamics of tumor–CD4+–cytokine–host cells interactions with treatments. Applied Mathematics and Computation. 2018;321:700–720.

11. Arciero J, Jackson T, Kirschner D. A mathematical model of tumor-immune evasion and siRNA treatment. Discrete & Continuous Dynamical Systems-B. 2004;4(1):39.

12. Makhlouf AM, El-Shennawy L, Elkaranshawy HA. Mathematical modelling for the role of CD4+ T cells in tumor-immune interactions. Computational and mathematical methods in medicine. 2020;2020.

13. Macallan DC, Asquith B, Irvine AJ, Wallace DL, Worth A, Ghattas H, et al. Measurement and modeling of human T cell kinetics. European journal of immunology. 2003;33(8):2316–2326.

14. Lee HY, Topham DJ, Park SY, Hollenbaugh J, Treanor J, Mosmann TR, et al. Simulation and prediction of the adaptive immune response to influenza A virus infection. Journal of virology. 2009;83(14):7151–7165.

15. de Pillis LG, Radunskaya AE. Modeling tumor–immune dynamics. In: Mathematical Models of Tumor-Immune System Dynamics. Springer; 2014. p. 59–108.

16. de Pillis LG, Gu W, Radunskaya AE. Mixed immunotherapy and chemotherapy of tumors: modeling, applications and biological interpretations. J Theor Biol. 2006;238(4):841–62. doi:10.1016/j.jtbi.2005.06.037.

17. Mclean AR, Michie CA. In vivo estimates of division and death rates of human T lymphocytes. Proceedings of the National Academy of Sciences. 1995;92(9):3707–3711.

18. Ribeiro RM, Mohri H, Ho DD, Perelson AS. In vivo dynamics of T cell activation, proliferation, and death in HIV-1 infection: why are CD4+ but not CD8+ T cells depleted? Proceedings of the National Academy of Sciences. 2002;99(24):15572–15577.

19. Rhodes A, Hillen T. A mathematical model for the immune-mediated theory of metastasis. Journal of theoretical biology. 2019;482:109999.
